# Prevalence of compassion fatigue among post-graduate trainees in Pakistan: A cross-sectional study

**DOI:** 10.12669/pjms.40.9.9265

**Published:** 2024-10

**Authors:** Saima Norin, Usman Mahboob, Naheed Mahsood, Sohail Raziq

**Affiliations:** 1 Saima Norin, BDS, MHPE (KMU). CMH Lahore, Pakistan; 2 Usman Mahboob, MBBS, MPH, DHPE, Post-Doc, Fellow FAIMER. Institute of Health Professions Education and Research, Khyber Medical University, Peshawar, Pakistan; 3 Naheed Mahsood, MBBS, MHPE. Department of Medical Education, Khyber Girls Medical College, Peshawar, Pakistan; 4 Sohail Raziq, MBBS, FCPS (General Surgery), FRCS (Urol). Department of Urology, CMH Lahore, Pakistan

**Keywords:** Burnout, Compassion fatigue, Compassion satisfaction, Postgraduate trainees, Professional quality of life

## Abstract

**Objective:**

To determine the prevalence of compassion fatigue, compassion satisfaction and burnout and identify the socio-demographic and work-related factors associated with compassion fatigue among FCPS Part-II trainees in Pakistan.

**Method:**

A cross-sectional study of FCPS-II trainees was conducted using stratified sampling at multiple centers over six months. Data was collected through an online, self-administered survey, which consisted of the 30-item ProQOL-V and a socio-demographic and work-related characteristics questionnaire. Informed consent was obtained from all participants. Data was analysed using SPSS 26 for descriptive stats, one-sample t-test, Pearson correlation, and multivariate linear regression at a 95% significance level.

**Results:**

Out of 460, only 392 trainees completely filled the online survey (completion rate: 90.74%). The study found that 78.80% of postgraduate trainees experienced moderate levels of compassion fatigue (CF) (Mean = 27.6, SD = 6.3), with moderate burnout (BO) (75.50%, Mean = 26.6, SD = 5.9), while moderately high Compassion satisfaction (CS) (90.60%, Mean = 33.3, SD = 5.5). Compared to normative data, compassion fatigue levels were significantly elevated among post-graduate trainees (p < 0.001). Significant correlations were observed between CF and BO (r =0.59), CF and CS (r = -0.20), and BO and CS (r = -0.63). Factors associated with higher CF included family dependents (p = 0.029), longer working hours (p < 0.001), and inadequate sleep (p < 0.001). Trainees in “Poor” work environments reported higher CF levels than those in “Excellent” environments (p < 0.001). Additionally, engaging in self-care activities such as exercise, prayer, and socialising were associated with lower CF levels (p < 0.05).

**Conclusion:**

The study revealed that many postgraduate trainees experience moderate compassion fatigue and burnout, with a strong positive correlation between CF and BO. Compassion satisfaction inversely related to both CF and BO, highlighting the need to boost CS. Factors like longer work hours, poor sleep, family dependents and unfavorable work conditions were linked to higher Compassion Fatigue. Conversely, engaging in self-care practices like prayer, meditation, exercise, and socialising is associated with decreased compassion fatigue levels. These results stress the importance of tailored interventions to enhance trainees’ well-being and ultimately improve patient care quality.

## INTRODUCTION

Empathy and compassionate care are essential for healthcare providers, including postgraduate trainees, to foster therapeutic relationships with their patients. However, their demanding and stressful work can affect their well-being, leading to *compassion fatigue (CF)*, negatively impacting their ability to deliver high-quality care.^1^ It is caused by consistent exposure to patients experiencing illness or trauma, leading to physical and mental exhaustion and reducing caregivers’ ability to empathise.^2^

According to Stamm, *Compassion* has two facets - *compassion satisfaction* (CS) and *compassion fatigue* (CF). CS can give a sense of satisfaction and joy, whereas CF negatively affects the helper’s personal and professional life. CF includes burnout and secondary traumatic stress (STS), which is driven by work-related trauma, which can be primary, secondary, or a mix of both.^2^,^3^

Often, healthcare providers with CF are unaware of their condition, causing them to suffer and may experience much suffering.^3^ If left unaddressed, it can lead to negative effects on their well-being, such as poor morale, depression, strained relationships, addiction, and more. CF can also cause organisational consequences such as higher staff turnover, medical errors, and diminished productivity.^4^-^6^

Postgraduate training can be stressful due to long working hours, increased workload, frequent rotations, lack of autonomy, role ambiguities, sleep deprivation and constant exposure to human suffering.^6^ Although CF is well-studied in nurses, psychiatrists, and physicians^7^,^8^, little is known about its prevalence among postgraduate trainees, particularly in low- and middle-income countries such as Pakistan.^7^ This knowledge gap may impact healthcare outcomes for patients.

## METHODS

This study used a quantitative, cross-sectional survey, over six months, to investigate the prevalence and factors associated with CF among post-graduate trainees at the PGMI-Rawalpindi and PGMI-Peshawar, accredited by The College of Physicians and Surgeons Pakistan (CPSP) for providing training in FCPS part-II.

### Ethical Approval:

It was obtained from Khyber Medical University’s Advance Study and Research Board on May 31^st^, 2023 (Ref. No. ASRB002053/CF/IHPE) and Ethical Review Board on June 13^th^, 2023 (Ref No.1-11/IHPER/MHPE/KMU/23-22). Online informed consent was obtained from all participants.

### Inclusion and Exclusion Criteria:

The study included FCPS trainees registered with CPSP, using a stratified random sampling technique based on specialty. The initial sample size was 383, due to online data collection, it was increased by 17% to a total of 460. Exclusion criteria involved trainees with less than six months of training or those on a training break.

The study used the ProQOL-V to measure CF, BO and CS. This self-report tool consists of 30 items categorised into three subscales. Scoring was done using a 1-5 Likert Scale with specific item inversion, with a total score of 22 or less indicating low rating, 23-41 points average, and 42 or more signifying a higher level. The ProQOL-V has good reliability (Cronbach’s α: CS = 0.88, BO = 0.75, STS = 0.81) and construct validity. The pilot study with 30 participants, confirmed the instrument’s reliability and validity. In addition to ProQOL-V, socio-demographic and work-related data were collected from participants.

This study collected a dataset from trainees and analysed using SPSS version 26.0. ProQOL-V questionnaires were scored and analysed as continuous and ordinal data in three categories: high, average, and low levels of CF, CS, and BO. Cronbach’s alpha gauged internal reliability, while normality was assessed through kurtosis, skew, and histogram analysis. A one-sample t-test was used to assess statistical differences between the study sample and normative data on CF, BO and CS using criteria from Seemann et al. (2019).^3^ Pearson’s correlation coefficient was utilised to analyse correlations between subscales, while Multivariate linear regression was used to evaluate the links between socio-demographic/work-related factors and CF. Statistical significance was set at p<0.05, with 95% confidence intervals.

## RESULTS

A total of 460 questionnaires were distributed and 435 responses were received, achieving a 94% response rate. Three hundred ninety-two (392) individuals completed the survey, yielding a completion rate of approximately 90.74%. Forty-three responses were excluded due to incomplete data.

**Fig.1:**
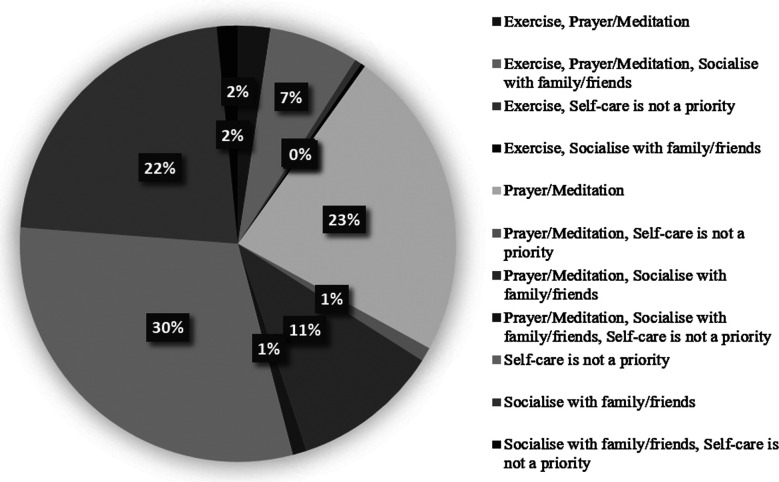
Self-care Priorities for Well-being

**Fig.2:**
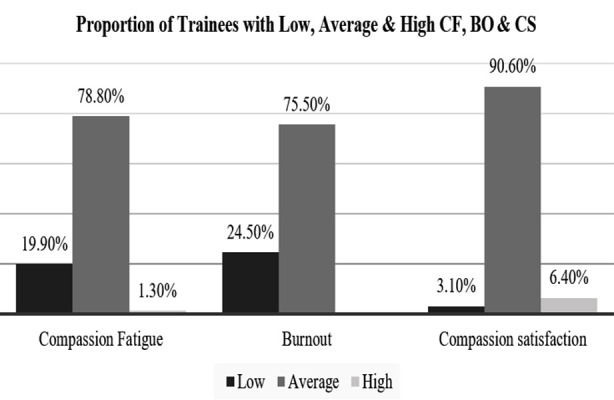
Distribution of Compassion Fatigue, Burnout, and Compassion Satisfaction among Trainees.

In Table-I the socio-demographic and work-related characteristics of post-graduate trainees are presented. The majority were male, aged 24-30, married, and had family dependents. Most worked >8 hours/day, on rotating shifts, and slept <6 hours/day. The largest earning range was >90,000 PKR per month, capping below 110,000 PKR. The study found that prayer/meditation, socialising, and exercise were the most common self-care activities; however, the majority did not prioritise self-care (Fig.1). Participants reported moderate levels of CF and BO, and moderate to high levels of CS, as detailed in Table-II-a with corresponding mean scores and standard deviations (Fig.2). The internal consistency measured for CF, BO, and SC subscales were high, with values of 0.81, 0.79, and 0.87, respectively.

**Table-I:** Socio-demographic and Work-related Characteristics of Trainees (N= 392).

Characteristics	n (%)
***Gender***	
Female	153(39)
Male	239(61)
***Age (in years)***	
24-30	224(57.1)
31-35	123(31.4)
36-40	31(7.9)
41-49	14(3.6)
***Marital status***	
Married	262(66.8)
Single	130(33.2)
***Current household members***	
Joint Family System	186(47.4)
Lives alone	63(16.1)
Spouse	37(9.4)
Spouse and Child(ren)	106(27.0)
***Family dependents***	
No	152(38.8)
Yes	240(61.2)
***Year of training***	
1st	111(28.3)
2nd	79(20.2)
3rd	56(14.3)
4th	109(27.8)
5th	37(9.4)
***Average working hours/day***	
< 8 h	79(20.2)
> 8 h	313(79.8)
***Work shifts predominantly***	
Day	136(34.7)
Night	3(0.8)
Rotating	253(64.5)
***Sleeping hours/day***	
< 6 h	234(59.7)
> 6 h	158(40.3)
***Salary in thousands***	
30-50	64(16.3)
51-70	17(4.3)
71-90	74(18.9)
More than 90(Capping Below 110,000PKR)	237(60.5)
***Workplace environment***	
Excellent	33(8.4)
Very good	88(22.4)
Good	211(53.8)
Poor	60(15.3)
***Use smoking products***	
No	342(87.2)
Yes	50(12.8)

**Table-II:** Study objectives for the prevalence of compassion fatigue among post-graduate trainees

(Objective-1) II-a: Prevalence of CF, BO and CS (One-Sample t-Test Using Normative Data from^3^									

Subscale	Sample Mean (SD)	Normative Mean (SD)			p-Value			95 %CI	
CS	33.3 (5.5)	36.9 (6.7)			< 0.001			[-4.1, -3.07]	
CF	27.6 (6.3)	21.2 (6.3)			<0.001			[5.76, 7.01]	
BO	26.6 (5.9)	26.2 (5.6)			0.17			[-0.18, 1]	

***(Objective-2) II-b:*** *Multivariate linear regression analysis to evaluate the potential predictors of CF, BO & CS.*									

*Variable*	*Category*		*CF*			*BO*		*CS*	

			*β*	*p-value*		*β*	*p-value*	*β*	*p-value*

Gender	Female (Reference)								
	Male		-0.09	0.078		-	-	-	-
Age	24-30 (Reference)								
	31-35		-	-		-	-	-	-
	36-40		-	-		-	-	0.126	0.015
	41-49		-	-		-0.14	0.007	-	-
Marital status	Married (Reference)								
	Single		-	-		-	-	-	-
Current Household members	Joint family system (Reference)								
	Lives alone		-1.10	0.059		-	-	-	-
	Spouse		-	-		-	-	-	-
	Spouse & child(ren)		-	-		-	-	-	-
Family dependents	No (Reference)								
	Yes		0.11	0.029		-	-	-	-
Year of training	1^st^year (Reference)								
	2^nd^ year		-	-		0.125	0.035	-	-
	3^rd^ year		-	-		-	-	-	-
	4^th^ year		-	-		-	-	-	-
	5^th^year		-	-		-	-	-	-
Work shifts	Day (Reference)								
	Night		-	-		-	-	-	-
	Rotating		-	-		0.141	0.006	-	-
Average working hours/day	-		0.19	< 0.001		0.243	<0.001	-0.103	0.042
Sleeping hours/day	More than 6 hours (Reference)								
	Less than 6 hours		0.20	< 0.001		0.157	0.002	-	-
Workplace environment	Excellent (Reference)								
	Good		0.27	0.003		0.467	<0.001	-0.447	<0.001
	Poor		0.34	<0.001		0.578	<0.001	-0.529	<0.001
	Very good		-	-		-	-	-.0192	0.015
Salary per month (in thousands)	30-50 (Reference)								
	51-70		-	-		-	-	-	-
	71-90		-	-		-	-	-	-
	More than 90		-	-		-	-	-	-
Use smoking products	No (Reference)								
	Yes		-	-		0.109	0.031	-	-
Self-care activities	Self-care is not a priority (Reference)								
	Prayer/ meditation		-0.20	<0.001		-0.272	<0.001	0.187	<0.001
	Socialize with family/friends		-0.11	0.057		-0.258	<0.001	0.139	<0.001
	Prayer/Socialize		-	-		-0.185	<0.001	0.197	<0.001
	Exercise/ Prayer/ Socialize		-0.21	<0.001		-0.262	<0.001	0.188	<0.001
	Exercise		-0.10	0.059		-0.186	<0.001	0.199	<0.001

Dependent Outcome Variables= Compassion Fatigue (CF), Burnout (BO), Compassion satisfaction (CS) Standardised Coefficients (Beta) = β. *Significant at P<0.05.

The sample exhibited significantly higher levels of compassion fatigue and significantly lower levels of compassion satisfaction compared to the normative data. However, burnout did not exhibit a significant variance from the population mean (Table-II-a). A study found that CF and BO have a positive correlation (r = 0.59, p < 0.01), while BO and CS have a negative correlation (r = -0.63, p < 0.01). Additionally, CF and CS have a weak negative correlation (r = -0.20, p < 0.01).

The multivariate linear regression analysis found that longer working hours, less than six hours of sleep per night, and poor work environments compared to excellent ones were associated with higher compassion fatigue (p < 0.001 for all). Conversely, engaging in prayer/meditation and a combination of exercise, prayer, and socialising were linked to lower CF levels (p < 0.001). Having family dependents increased CF (p=0.029), while living alone was marginally associated with lower CF. Although not statistically significant, males had slightly lower CF levels than females (p=0.078). Age, marital status, years of training, work shift type, salary range, and smoking habits did not significantly affect CF (p > 0.05) (Table-II-b). In the analysis, burnout was significantly higher in second-year trainees, those with rotating shifts, longer working hours, less than six hours of sleep, and poor work environments. Self-care activities reduced burnout. Compassion satisfaction was significantly higher in individuals aged 36-40. Longer working hours and poor work environments decreased compassion satisfaction, whereas self-care activities increased it (Table-II-b).

## DISCUSSION

The study revealed that postgraduate medical trainees experience moderate levels of both compassion fatigue and burnout, despite having a moderate level of compassion satisfaction. This dual experience underscores the complex nature of trainee experiences in the demanding FCPS training program. The findings align with existing literature highlighting the increased stress and emotional toll associated with medical training.^3^,^6^,^9^,^10^ and also highlights that high CS does not prevent them from experiencing significant levels of CF.

Trainees in this study displayed significantly higher CF scores (27.6) than Western norms (p < 0.001), indicating heightened CF in the unique context of FCPS training.^3^ The study also revealed a lower quality of life for trainees than Canadians who took the same survey. This discrepancy suggests that the distinctive challenges and stressors of the FCPS program, as well as resource constraints and healthcare structure differences in developing countries, contribute to elevated CF levels beyond the general population.

Furthermore, the study uncovered that higher levels of CF are associated with increased BO.^8^ Conversely, the negative relationship between BO and CS suggests that elevated BO erodes the ability to find fulfilment in supportive roles.^11^,^12^ Additionally, increased CF may lead to lower job satisfaction among trainees.^13^ These findings highlight the need for interventions to mitigate CF and BO and enhance CS to improve the trainees’ well-being.

Living alone showed a marginal association with lower CF, indicating the potential benefits of independence and reduced interpersonal stressors. In contrast, having family dependents was significantly linked to higher CF levels, reflecting the additional emotional and practical responsibilities that may worsen stress among trainees.^6^,^14^ These findings illuminate the importance of social support and caregiving responsibilities in shaping CF experiences, calling for tailored support strategies to meet trainees’ diverse familial and living situations.

Getting sleep over six hours a day emerged as a protective factor against CF and BO, which aligns with previous research highlighting that sleep deprivation negatively impacts mental and physical health.^15^ Therefore, prioritising sleep is crucial to alleviate CF in trainees. Effective *self-care* practice demonstrated protective effects against CF and BO.^9^ The findings emphasise the need to promote healthy habits and self-care in medical training to build trainee resilience and reduce stress impact.

The study found that extended *work hours* were positively linked with increased CF and BO, reinforcing past findings.^6^,^8^ This underscores the detrimental impact of prolonged work hours on the well-being of postgraduate trainees, highlighting the need for interventions to address workload-related stressors and prioritise trainees’ work-life balance.

Workplace conditions have a significant effect on the levels of compassion fatigue and burnout experienced by trainees. Individuals working in “Good” and “Poor” conditions experienced elevated CF and BO, whereas, those in “Excellent” conditions were found to be protected, consistent with previous research.^16^ This highlights the pivotal role the *workplace environment* plays in reducing trainees’ psychological distress.

Gender’s non-significance (p = 0.078) in predicting compassion fatigue aligns with previous findings,^4^,^14^ yet males exhibit marginally lower CF levels, consistent with systemic reviews.^2^ The lack of association between CF and age or marital status challenges prior research,^14^ suggesting broader work-related and self-care factors influence CF more significantly. Age notably impacts compassion satisfaction and burnout, with older trainees (36+) reporting higher CS, indicating increased satisfaction with caregiving responsibilities.^17^,^18^ Younger individuals (24-40) have the lowest BO risk compared to those over 40.

No significant links were found between CF and work *shift type*, *salary range*, or *smoking product* use. However, the study revealed that the second year of training, rotating work shifts, and smoking product use were linked to higher levels of burnout among medical trainees.

Interventions targeting work-life balance, sleep hygiene, supportive work environments, and policy-making are crucial to mitigate compassion fatigue and burnout among medical trainees. Future research should include qualitative studies to explore the subjective experiences of trainees and longitudinal studies to track the long-term effects of interventions.

### Strengths and limitations:

This study offers a thorough analysis of compassion fatigue, burnout, and compassion satisfaction in postgraduate medical trainees, highlighting the importance of self-care and workplace conditions. However, it has limitations, including non-response bias, a cross-sectional design, and the inability to draw causal conclusions.

## CONCLUSION

Compassion fatigue and burnout are highly prevalent among post-graduate trainees, accompanied by an inverse relationship with compassion satisfaction, necessitating tailored interventions in training programs to enhance CS and mitigate the adverse effects of CF and BO. Strategies that promote, work-life balance, self-care and ample sleep should be integrated into training programs to enhance well-being. Creating supportive and healthy work environments is crucial, as workplace conditions play a significant role in CF. Gender-related trends in CF experiences require further investigation, along with potential psychosocial dynamics associated with living arrangements. This research serves as a foundation for further studies and interventions to reduce CF among post-graduate trainees.

### Authors Contribution:

**SN:** Conceived, designed, conducted, analysed and interpreted the study, drafted the manuscript and ensured content integrity. **UM:** Supervised and guided study design, critically reviewed and edited the manuscript. **NM:** contributed to the study design and manuscript review. **SR:** contributed to the study design, data collection and manuscript review. All authors approved the final manuscript.
